# Supplementary material for: Complexome profiling of the Chlamydomonas psb28 mutant reveals TEF5 as an early PSII assembly factor
Source: Plant Cell. 2025 Mar 17;37(6):koaf055. doi: 10.1093/plcell/koaf055 (PMC12164590; doi:10.1093/plcell/koaf055)
Supplement: koaf055_Supplementary_Data [file koaf055_supplementary_data.zip › psb28_interactive.zip!/plots/intensity/qualityPlots/peptides/ULP1_Cre01.g051500.t1.2.html]

### Cre01.g051500.t1.2

Trivial name: ULP1  
SeqCoverage [median]: 29.90  
Mass: 11.98 kDa  
  
 

| Sequence | MissedCleavage | Mass [Da] | wtIntensity | muIntensity | Score |
| --- | --- | --- | --- | --- | --- |
| IPSHEFTGGMVK | 0 | 1301.64 | 29602.29 | 0.00 | 98.90 |
| KQGVSLK | 1 | 758.47 | 17928.00 | 0.00 | 110.61 |
| SPTAASMESYTLEGTK | 0 | 1671.77 | 69472.90 | 4528.67 | 265.22 |
| SPTAASMESYTLEGTKK | 1 | 1799.86 | 520960.22 | 62474.71 | 284.53 |
| SPTAASMESYTLEGTKKQGVSLK | 2 | 2412.22 | 713.58 | 0.00 | 88.04 |
|  |  |  | 638677.0 | 67003.4 |  |
